# Depth-estimation of stiffness singularity in an elastic object via directional touch sensing using microfinger with tactile sensor

**DOI:** 10.1038/s41598-025-25774-y

**Published:** 2025-11-25

**Authors:** Y. Hori, S. Konishi

**Affiliations:** 1 Graduate School of Science and Engineering, Ritsumeikan University, Shiga, Japan; 2 Department of Mechanical Engineering, Ritsumeikan University, Shiga, Japan; 3 Ritsumeikan Advanced Research Academy, Kyoto, Japan; 4 Ritsumeikan Global Innovation Research Organization, Kyoto, Japan

**Keywords:** Medical research, Engineering

## Abstract

Understanding object information during robotic hand grasping is a key goal in robotics. Researchers have integrated tactile sensors to replicate artificial haptics on humanoid robot fingertips, but robotic grasping has yet to be fully applied in palpation-based medical diagnosis. Current techniques, such as vibration-based ultrasound-assisted surgeries, face limitations in diagnosis due to anatomical constraints or surgical access issues. To address this, we explored palpation-assisted surgeries using a microfinger, a miniaturized version of a human finger. We developed micromachine-based palpation techniques for advanced minimally invasive diagnosis using endoscopes. Specifically, we developed a microfinger with artificial muscle and tactile sensors, designed to detect stiffness singularities in pseudo-biological tissues. Our microfinger, thin and small, exerted a pushing force greater than 1 N and performed directional palpation. Next, we proposed an algorithm for estimating three-dimensional coordinates, thus transcending the existing two-dimensional singularity-estimation method. Consequently, we achieved touch sensing on silicone gel blocks using a small rigid ball, with depth-estimation of approximately ± 1.3 mm at a depth of 15 mm. The directivity of the microfinger enabled three-dimensional positional estimation of the singular point. We present a breakthrough for microfinger-based palpation technology for medical diagnosis, accelerating the advancement of robotics-based palpation-driven minimally invasive techniques.

## Introduction

In robotics, tactile perception plays a key role in enabling accurate and controlled object handling. This also applies to minimally invasive surgery (MIS), where robotic systems have become increasingly common. Adding tactile sensing to the surgical tools is considered necessary to improve both accuracy and safety in such procedures. MIS, represented by laparoscopic procedures, has recently emerged as a major procedure owing to its potential to reduce the physical burden on patients because of smaller incisions. However, one major limitation of this approach is the lack of direct tactile interaction with internal organs^1^. In traditional open surgery, palpation was one of the methods used by surgeons to identify tumors, taking advantage of the fact that tumors are typically stiffer than surrounding tissue^2^. In contrast, MIS prevents direct touch with the lesion, as surgical instruments are inserted through narrow trocars. This absence of tactile sensation can lead to difficulties in accurately identifying tumor locations and may cause unintended tissue damage due to misapplied force during manipulation^3,4^. Consequently, providing tactile feedback has become a key research focus to enhance safety and precision in MIS.

To compensate for the lack of tactile feedback in MIS, tumor localization is typically supported by preoperative imaging and confirmed during surgery using ultrasound. However, accurate intraoperative tumor identification becomes difficult due to limitations in trocar-based insertion and restricted probe control, which hinder proper contact with organs and reduce the accuracy of detection^5^. Moreover, surgeons still desire tactile feedback similar to that provided by open surgery. These challenges highlight the need for the development of tactile-based tumor localization devices to complement ultrasound techniques.

Various tactile sensors and perception tools have been developed to simulate human touch^6–9^. As examples of tactile sensing technologies, electronic skin mimics the structure of human fingertips and enables simultaneous detection of slip and pressure^6^^.^ It is fabricated using carbon nanotube–polydimethylsiloxane (PDMS) composites and incorporates a triboelectric generator, porous microstructures for pressure sensing, and a fabric-based supercapacitor for power storage, allowing operation without external power sources. Other advancements include flexible dual-mode capacitive sensors capable of both proximity-based contactless sensing and pressure detection, suitable for wireless integration^7^. Additionally, Micro-electromechanical systems-based six-axis force–torque sensors have been embedded into grasping forceps to estimate the position and size of embedded objects—such as silicone balls inside gelatin phantoms—through force analysis^8^. A separate approach involves a noncontact stiffness imager, which applies force via fluid and evaluates the resulting displacement to estimate stiffness distributions, avoiding friction and reducing the risk of tissue damage^9^. Accurate depth estimation is also essential for effective tumor removal. Several approaches have been reported, including combining force and positional data^10^ or applying deep learning to analyze tissue deformation patterns^11,12^. These advancements suggest the potential of compact tactile devices capable of being inserted into the body through laparoscopic instruments.

**Fig. 1:**
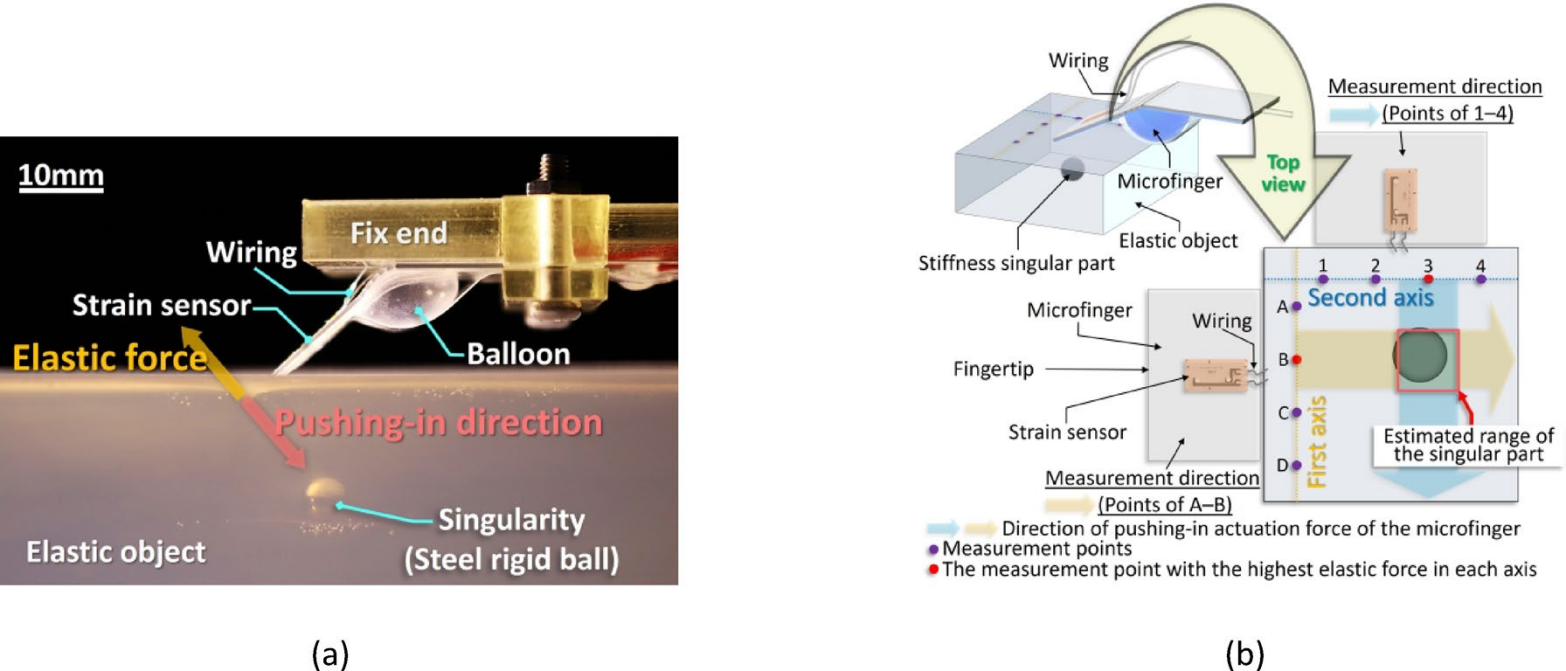
Searching for stiffness singularity by using directivity of the microfinger (**a**) This microfinger can evaluate the stiffness of the target by measuring the strain of the sensor integration area caused by the elastic force when pushing-in. In addition, since the bending motion is used for palpation, the microfinger has a directional property that enables detection of singularities in the push-in direction. (**b**) We have reported on the efficient searching for singularities using directivity of the microfinger^17^. The measurement was carried out by establishing two measurement axes and adjusting the bending direction of the microfinger to the axes. We have succeeded in estimating the location of the singularity on a two-dimensional surface.

Here, we achieved touch sensing by a microfinger with artificial muscles (pneumatic balloon actuators (PBAs)) and tactile sensors^13–18^. PBAs are composed of polymers, such as PDMS, and are characterized by their small sizes, flexibility, and safety. Further, we developed pneumatic bending actuators^19,20^ and designed a force-conversion film (Fig. 3a) to generate higher power^21–23^. The bending actuator was used to develop the microfinger for palpation in this study^17^. When pressure is applied, the balloon expands, and the force-conversion film is tensioned, thus bending the microfinger. The elastic force exerted by an object being pushed-in (Fig. 1a) strains the sensor-integration area, changing the sensor value, which provides information about tissue stiffness. We have already reported a method for searching for stiffness singularities in elastic objects using the microfinger^17^ One of the key features of this microfinger is its directivity, a sensing property observed during bending-based pushing-in motion. Although stiffness is generally evaluated through vertical indentation, our approach evaluates stiffness using a bending-actuated microfinger, which enables the acquisition of direction-dependent information by varying the pushing direction. The singularity in an elastic object is strongly detected when a vertical indentation is applied directly over it; however, utilizing directivity allows the detection of the singularity not only from directly over it but also from a distant position in front of the target (Fig. 1a). We proposed a method for searching for singularities; this method utilizes the directivity of the microfinger^17^. Figure 1b outlines a two-dimensional localizing algorithm that can efficiently estimate the singularity location. First, two measurement axes are established for the range, which is determined based on preoperative imaging, within which the singularity is assumed to exist (Fig. 1b). Thereafter, the microfinger is adjusted along the first axis so that its bending direction faces the inside of the range, followed by the measurement of points (A–D). As shown in Fig. 1b, the singularity can be estimated to exist on the extension of the bending direction of measurement point B. In the next step, the bending direction of the microfinger is aligned with the second axis, and the measurement point (1–4) is measured. The singularity can be estimated to exist at the extension of point 3. From the results of the two-axis measurements above, it can be estimated that the singularity is located at the intersection of the extension of points B and 3. This approach allowed for the efficient estimation of the coordinates of the singularity in the two-dimensional surface^17^.

## Results and discussion

### Three-dimensional localizing algorithm

**Fig. 2:**
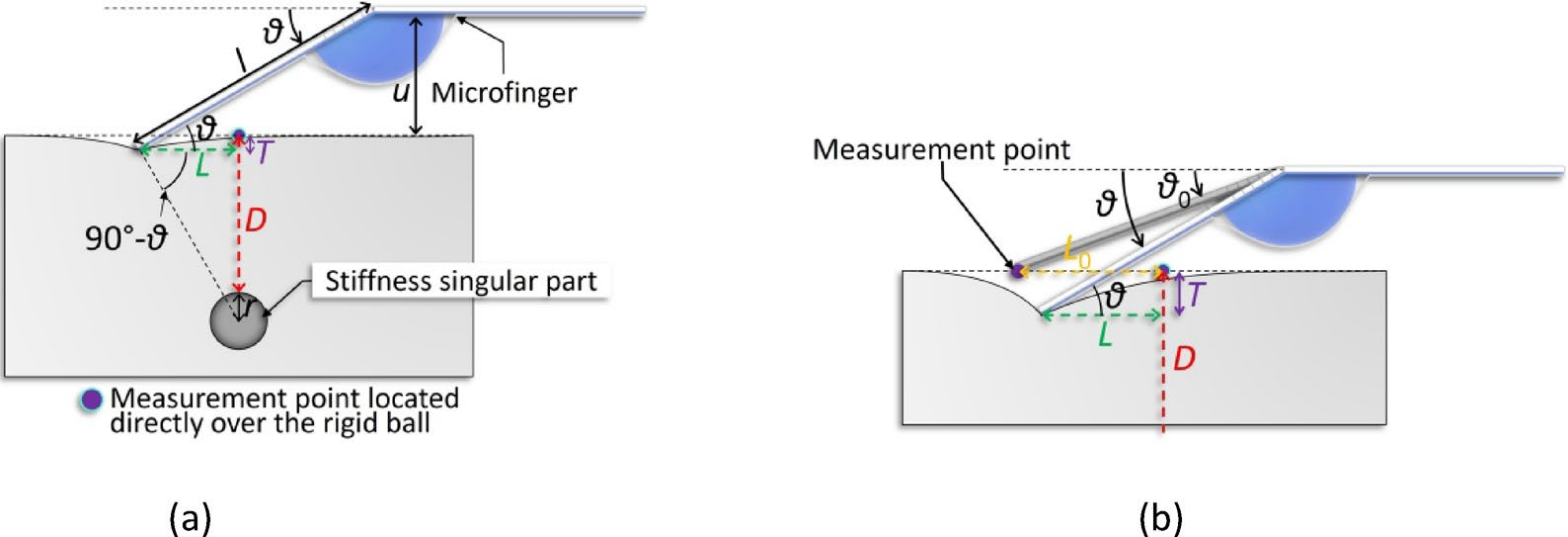
Three-dimensional localizing algorithm for stiffness singular part (**a**) It is considered that the singularity is located in the extension of the pushing-in direction when the microfinger detects the singularity from the front position. (**b**) The tip of the microfinger contacts at the measurement point during palpation. The tip position after pushing-in (*θ*) moves in the singularity side by ${\it{\text{L}}}_{{0}} - {\it \text{L}}$.

Our two-dimensional localizing algorithm efficiently estimates the singularity’s plane coordinates. This study proposes a three-dimensional localizing algorithm to estimate singularity depth, using the two-dimensional algorithm and the microfinger’s directivity, assuming tumors located at depths of up to approximately 15 mm. The three-dimensional localizing algorithm was based on the assumption that the coordinates directly over the singularity were known by the two-dimensional localizing algorithm. It requires measuring multiple points in one dimension passing directly over the singularity to determine the point where the elastic force increases. The singularity is assumed in the microfinger’s pushing-in direction when the elastic force increases (Fig. 2a). Figure 2a also shows the geometric relationship when the microfinger’s pushing-in force balances with the object’s elastic force at the measurement point. At this point, the microfinger operates horizontally with respect to the target plane. Figure 2b shows the microfinger tip position difference when it contacts the measurement point and when it finishes pushing in and is balanced. Each symbol represents the following: $\it {\uptheta }$, bending angle (contact angle) in the balanced state after pushing-in; ${\it {\uptheta }}_{{0}}$, bending angle (contact angle) at the moment of contact with the target; $\it {\text{u}}$, the distance between the microfinger and target; $\it {\text{r}}$, radius of the singularity; $\it {\text{l}}$, length from the bending point of the microfinger to the contact point; $\it {\text{T}}$, the amount of pushing-in by the microfinger on the target; $\it {\text{D}}$, singularity depth; and $\it {\text{L}}$, the distance between the area directly over the singularity and contact point of the microfinger after pushing-in. Moreover, the pushing-in amount $\it {\text{T}}$ can be derived from the amount of change in the bending angle from the moment the microfinger contacts the target until the end of the pushing-in, as follows:

1 $$\mathit{T} = \mathit{l}\left( \sin \mathit{\theta} - \sin \mathit{\theta}_{0} \right)$$

The relationship between $\it {\text{u}}$ and ${\it {\uptheta }}_{{0}}$ is expressed, as follows:

2 $$u = l\,\mathrm{sin}\,\theta_{0}$$

Afterward, $\it {\text{L }}$ was considered. As depicted in Fig. 2b, $\it {\text{L }}$ changed before and after applying the pushing-in motion. During the measurement, the elastic force was measured assuming that the pushing-in actuation force and elastic force were in equilibrium. Conversely, the measurement point was based on the initial position before pushing in, and this caused a difference in $\it {\text{L }}$(${\it {\text{L}}}_{{0}}$). Thus, ${\it {\text{L}}}_{{0}}$ is defined as the distance between the measurement point directly over the singularity and the measurement point of the increase in the elastic force. Further, Fig. 2b shows the relationship between $\it {\text{L}}$ and ${\it {\text{L}}}_{{0}}$. Both parameters can also be calculated from the amount of change in the bending angle from the moment the microfinger contacts the target until the end of the pushing-in, as follows:

3 $$L = L_{0} - l\;\left( {\cos \theta_{0} - \cos \theta } \right)$$

Finally, the $\it {\text{D}}$ was calculated. As shown in Fig. 2a, the angle between the lines of pushing-in direction and $\it {\text{L}}$ is given by ${90}^{ \circ } - \it {\uptheta }$, and the following relationship can be derived:

4 $${ }D + r - T = L\;\tan (90^{ \circ } - \theta )$$

(However, *T* < *D*)

Substituting Eqs. 1 and 3 into 4 to derive $\it {\text{D}}$ yields the following:

5 $$D = \frac{{L_{0} - l(\cos \theta_{0} - \cos \theta )}}{\tan \theta } + l (\sin \theta - \sin \theta_{0} ) - r$$

We explored the possibility of estimating $\it {\text{D}}$ using Eq. 5. Notably,${\it {\text{L}}}_{0}$ can be determined from the distance between the point directly over the coordinates obtained by the two-axis measurement algorithm and the measurement point where the elastic force is increased, $\it {\text{l}}$ represents the microfinger dimension, and ${\it\uptheta }_{{0}}$ can be derived from Eq. 2. Therefore, $\it {\text{D}}$ can be estimated if $\it {\text{r}}$ is known in advance through imaging techniques or other methods, and the information on $\it {\uptheta }$ during the application of the pushing-in actuation can be obtained.

### Evaluation of the three-dimensional localizing algorithm

**Fig. 3:**
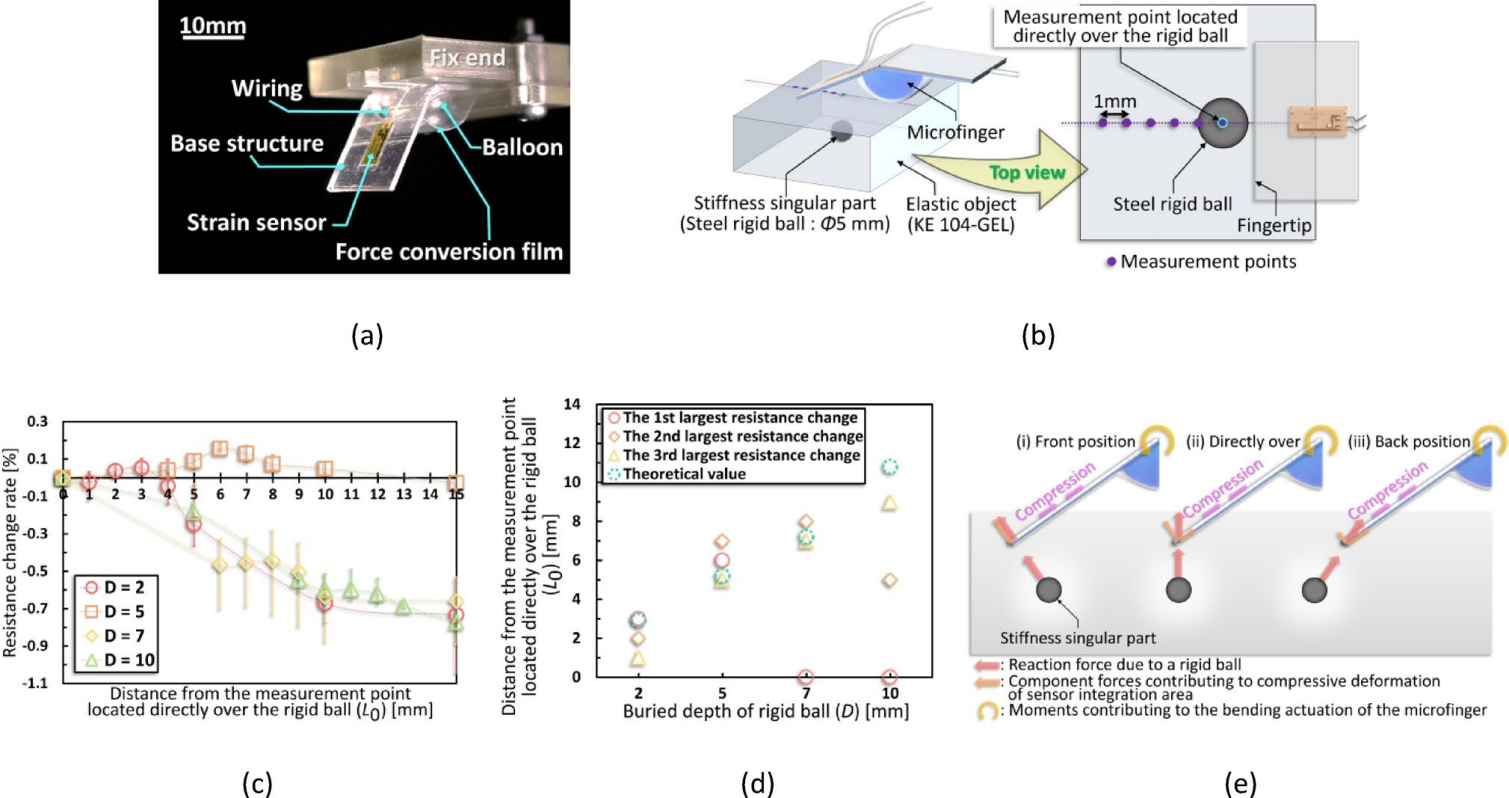
Evaluation of three-dimensional localizing algorithm (**a**) Appearance of microfinger used in this evaluation. (**b**) Models with a rigid ball of 5 mm diameter buried as the singularity were used for the evaluation. Four depths of rigid balls (2, 5, 7, and 10 mm) were used for the evaluation. Measurements were carried out from a distance of 15 mm maximum, setting the position directly over the rigid ball as 0 mm. (**c**) For each singularity buried depth* D*, the resistance change rates with respect to the distance from the measurement point directly over the singularity is shown. (**d**) The distance from directly over the singularity is shown for each buried depth *D* in Fig. 3c. The locations of the three measurement points where the resistance change rate was the largest are shown. The theoretical values are also shown by substituting each parameter at the evaluation into Eq. 5. (**e**) The direction of the force received from the singularity varies depending on the pushing-in position of the microfinger with respect to the singularity. However, in any positions, the received force from the singularity contributes to the compressive deformation of the sensor-integration area, which increases the resistance change rate of the sensor.

As shown in Fig. 3a, a microfinger (16 × 40 × 0.965 mm) was used for evaluation. Further, Fig. 3b shows a small rigid steel ball with known radius *r*, used as the singularity. One-dimensional measurements were conducted through the point directly over the singularity. These measurements were performed in the 0–15 mm range in front of the singularity in the bending direction at 1 mm intervals around the measurement point where the elastic force presumably increased (this point was calculated in advance from $\it {\uptheta }$ and *D* based on Eq. 5) and at 5 mm intervals for the other ranges to obtain a rough distribution of the entire measurement range. Four depth conditions (*D* = 2, 5, 7, and 10 mm) were tested using a 5 mm diameter isotropic steel ball. The applied pressure was kept constant for each depth condition and controlled such that the pushing-in amount *T* was lower than *D*.

Figure 3c depicts the evaluation results. The horizontal axis represents ${\it\text{L}}_{{0}}$, the distance from each measurement point to the point directly over the ball, while the vertical axis shows the resistance change rate of the tactile sensor, normalized to the value at ${\it\text{L}}_{{0}}$ = 0 mm. As shown in Fig. 3d, the measurement points with the highest resistance change rates are plotted for each *D*. For reference, the second and third highest resistance-change points are also plotted. For example, under *D* = 5 mm, the maximum resistance change occurred at ${\it\text{L}}_{{0}}$ = 6 mm, followed by 7 mm and 5 mm. These values were plotted as *D* = 5 mm versus ${\it\text{L}}_{{0}}$ = 5, 6, and 7 mm in Fig. 3d. Green plots in Fig. 3d represent the theoretical values of elastic force increase, which are defined as ${\it\text{L}}_{{0}}$ derived by substituting the corresponding parameters into Eq. 5. The calculation used the buried depth of the rigid ball *D*, the dimension of the microfinger *l*, the reference angle ${\it\uptheta }_{{0}}$ derived from the measured displacement *u* using Eq. 2, the measured bending angle *θ* during evaluation, and the radius of the rigid ball *r*. Assuming the theory was correct, these plots should closely match the measured points of increased resistance. The measured values were obtained at 1 mm intervals, and the theoretical values reflected the 0.1 mm order. In Fig. 3c, for *D* = 2 and 5 mm, increased resistance was clearly observed at measurement points offset from ${\it\text{L}}_{{0}}$ = 0 mm, matching theoretical predictions (Fig. 3d). In contrast, for *D* = 7 and 10 mm, resistance increases were only slightly higher near the theoretical points, and in general, resistance tended to rise as measurements approached ${\it\text{L}}_{{0}}$ = 0 mm. Thus, the measured distribution diverged more from the theoretical one under these deeper conditions.

Figure 3e illustrates the increase in elastic force due to the singularity during pushing-in and shows the microfinger positions when its actuation force balances the elastic force at three locations: (i) in front of the ball, (ii) directly over it, and (iii) behind it. Although only region (i) was measured in this study, if *T* is large, the microfinger tip would stop behind the ball after pushing-in. Figure 3e shows that in (i), the reaction force received from the rigid ball causes compressive stress at the sensor-integration area, decreasing the resistance value. For (ii), the compressive stress acts on the sensor-integration area owing to the component force of the upward reaction force from the rigid ball, causing a decrease in the resistance value. For (iii), the direction of the reaction force due to the rigid ball coincides with the direction of the compressive stress on the sensor-integration area, causing a decrease in the resistance value. Notably, (iii) represents a scenario that holds only for *D* = 7, 10 mm (the conditions under which the rigid balls are deeply buried). The linear distance between the contact point of the microfinger and the rigid ball is the furthest under condition (i). Contrarily, the resistance change in (i) is assumed to be larger than that in (ii), even at farther distances, as the reaction force in (i) is directly converted into compressive stress, whereas that in (ii) is converted in a portion of the reaction force into compressive stress. However, as *D* of the rigid ball increases, the contact point between the microfinger and rigid ball moves further apart from each other, and the influences of (ii) and (iii), which are closer in linear distance than (i), dominate. Consequently, the resistance changes significantly in the front of the ${\it\text{L}}_{0}\text{ = 0 mm}$ when *D* = 2 and 5 mm (Fig. 3c), although the influences of (ii) and (iii) increase at* D* = 7 and 10 mm, respectively, allowing the resistance change rate to increase as it approaches the measurement point directly over.

### Protrusion design of the contact part to improve estimation accuracy

**Fig. 4:**
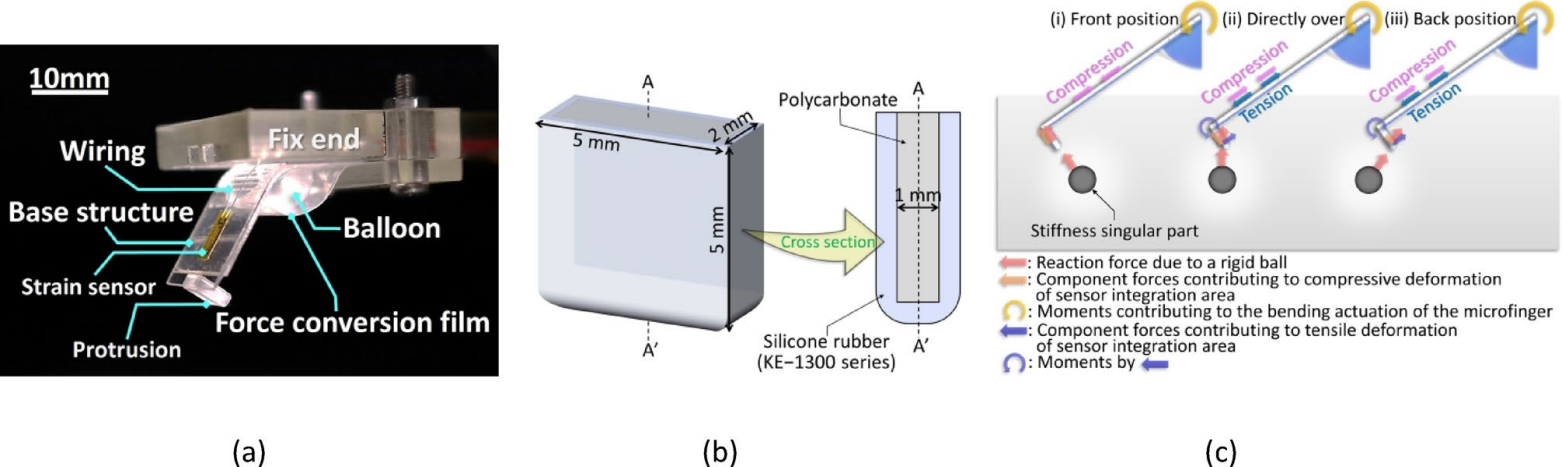
Integration of a protrusion on the contact area of the microfinger (**a**) Appearance of the protrusion-integrated microfinger. (**b**) Protrusion dimensions. (**c**) Compared to Fig. 3e, the sensor-integration area at positions (ii) and (iii) are also under tension stress, which is thought to reduce the amount of change in resistance. As a result, only the resistance change in position (i) is more noticeable, and the directivity is considered to be enhanced.

The results reported in Fig. 3c reveal that the depth-estimation accuracy decreased for deeper singularities. To overcome this issue, we designed a protrusion (Fig. 4b) and integrated it at the microfinger’s contact point. Figure 4a shows the protrusion-integrated microfinger.

Figure 4c shows (i) the front of the rigid ball in the bending direction, (ii) the measurement position directly over the rigid ball, and (iii) the back of the rigid ball, as in Fig. 3e. Following Fig. 4c, for (i), the reaction force from the rigid ball acts on the sensor-integration area as compressive stress, decreasing the resistance value. For (ii), the protrusion structure is acted on by the upward reaction force of the rigid ball, generating a moment at the fixed end of the protrusion structure as the rotation axis, causing tensile stress at the sensor area. Therefore, the decrease in the resistance is suppressed. Even in (iii), the reaction force due to the rigid ball acts on the protrusion, and the sensor-integration area is tensioned by the moment generated at the fixed end of the protrusion as the rotation axis, suppressing the decrease in the resistance value. The sensor resistance would only decrease in (i), whereas the decreasing resistance would be suppressed in (ii) and (iii), and the directivity would be enhanced by the integrated protrusion structure. Thus, deeper singularities could be estimated.

Further, Increasing the protrusion height reduces *θ* and increases the actuation force (Fig. 6a), improving detection when $\it {\text{u}}$ (Fig. 2a) is constant. For this evaluation, the height was set to 5 mm. The width dimension was also considered a related parameter to the scanning range and detection resolution (Fig. 6b, c). In our previous study^17^, we scanned the entire range using the microfinger at 5 mm measurement intervals (Fig. 1b) with respect to a rigid ball exhibiting a 5 mm diameter. Accordingly, the width of the protrusion was set as 5 mm to suit the 5 mm rigid ball in this case. The thickness was set such that the protrusion would not be deformed by the pushing-in actuation force.

### Three-dimensional localizing algorithm for the microfinger integrated with the protrusion structure

**Fig. 5:**
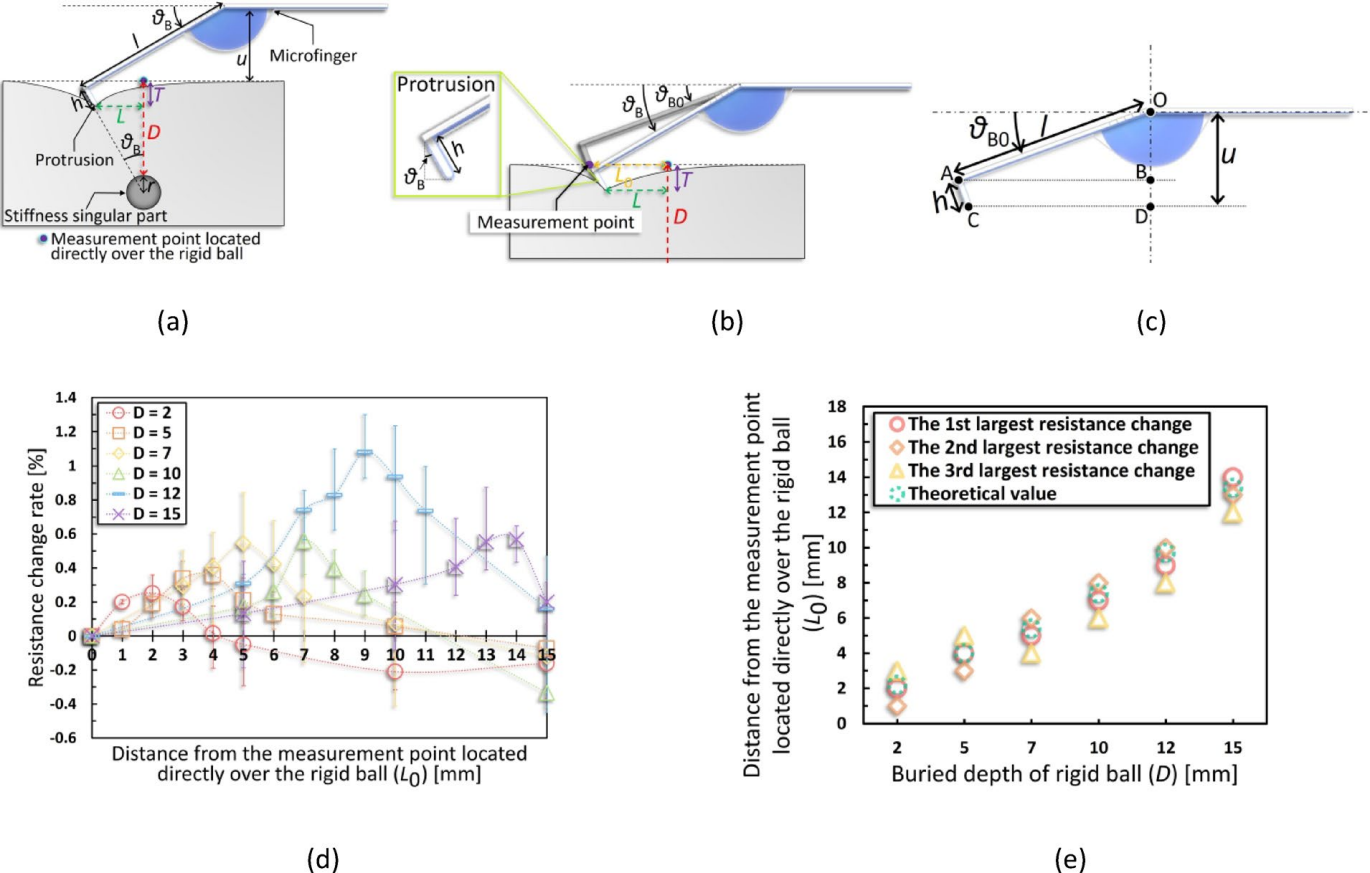
Three-dimensional localizing algorithm by microfinger with the protrusion structure (**a**) Positional relationship when there is a protrusion. (**b**) Shift of fingertip position when there is a protrusion. (**c**) Derivation of the bending angle ${\it\uptheta }_{{{\text{B0}}}}$ of the microfinger with protrusion when it contacts the target. (**d**) The same evaluation as in Fig. 3c was conducted for the case with the protrusion. The peaks were clearer due to the enhanced directivity by the protrusion. (**e**) Same as Fig. 3d, a comparison with the theoretical value is also shown, substituting each parameter during the evaluation in Eq. 9.

Next, we derived a theoretical equation for depth-estimation using the protrusion-integrated microfinger. Figure 5a illustrates the geometric model of this structure pushing- into a target at the measurement point where the elastic force increases, and the pushing-in actuation force and elastic force are in equilibrium. Here, ${\it\uptheta }_{{\text{B}}}$ represents the bending angle (contact angle) in the equilibrium state after pushing in, ${\it\uptheta }_{{{\text{B0}}}}$ represents the bending angle (contact angle) at the moment of contact with the target, and *h* represents the protrusion height. The other definitions are the same as those defined in Fig. 2. As shown in Fig. 5b, can be described by the following equation considering the change in ${\it\uptheta }_{{{\text{B0}}}}$ until the end of the pushing in, as in Eq. 1, and considering the amount of change due to the protrusion structure.

6 $$\mathit{T} = \mathit{l}\,(\sin \mathit{\theta}_{\mathrm{B}} - \sin \mathit{\theta}_{\mathrm{B0}}) + \mathit{h}\,(\cos \mathit{\theta}_{\mathrm{B}} - \cos \mathit{\theta}_{\mathrm{B0}})$$

The relationship between $\it {\text{L}}$ and ${\it\text{L}}_{{0}}$ is also derived in the same manner as in Eq. 3 considering the change due to the protrusion structure.

7 $$L = L_{0} - \{ l (\cos \theta_{\mathrm{B0}} - \cos \theta_{\mathrm{B}} ) - h (\sin \theta_{\mathrm{B0}} - \sin \theta_{\mathrm{B}} )\}$$

Next, we derived $\it {\text{D}}$. Figure 5a shows that the angle between the lines *D* and the pushing-in direction is ${\it\uptheta }_{{\text{B}}}$. Thus, the following relationship can be derived:

8 $$(\mathit{D} + \mathit{r} - \mathit{T})\,\tan \mathit{\theta}_{\mathrm{B}} = \mathit{L}$$

Thus, we obtained the following equation by substituting Eqs. 6 and 7 into Eq. 8.

9 $$\mathit{D} = \frac{\mathit{L}_{0} - \{\, \mathit{l} (\cos \mathit{\theta}_{\mathrm{B0}} - \cos \mathit{\theta}_{\mathrm{B}}) - \mathit{h} (\sin \mathit{\theta}_{\mathrm{B0}} - \sin \mathit{\theta}_{\mathrm{B}})\, \}}{\tan \mathit{\theta}_{\mathrm{B}}} + \mathit{l} (\sin \mathit{\theta}_{\mathrm{B}} - \sin \mathit{\theta}_{\mathrm{B0}}) + \mathit{h} (\cos \mathit{\theta}_{\mathrm{B}} - \cos \mathit{\theta}_{\mathrm{B0}}) - \mathit{r}$$

Equation 9 allows for the estimation of the depth information of singularities using the protrusion-integrated microfinger. Here, ${\it\uptheta }_{{\text{B}}}$ was determined by measuring the bending angle during pushing in, whereas ${\it\uptheta }_{{{\text{B0}}}}$ can be calculated from the dimensions of the microfinger and $\it {\text{u}}$.

The following explains the procedure for calculating ${\it\uptheta }_{{{\text{B0}}}}$. Figure 5c illustrates a model in which the protrusion-integrated microfinger is in contact with an object and where the bending angle is ${\it\uptheta }_{{{\text{B0}}}}$. In this case, the following two equations can be obtained because $\angle {\text{OAB}} = {\it\uptheta }_{{{\text{B0}}}}$.

10 $$\mathrm{OB} = \mathit{l}\sin \mathit{\theta}_{\mathrm{B0}}$$

11 $$\mathrm{BD} = \mathit{h}\cos \mathit{\theta}_{\mathrm{B0}}$$

Considering that OB + BD = $\it {\text{u}}$ holds, the following equation can be derived from 10 and 11.

12 $$\sqrt {l^{2} + h^{2} } \sin \left( {\theta_{\mathrm{B0}} + \alpha } \right) = u$$

$$({\text{However}},\cos \alpha = \frac{l}{{\sqrt {l^{2} + h^{2} } }} {\text{holds}})$$

Further, Eq. 12 can be rewritten, as follows.

13 $$\theta_{\mathrm{B0}} = \arcsin \left( {\frac{u}{{\sqrt {l^{2} + h ^{2} } }}} \right) - \arccos \left( {\frac{l}{{\sqrt {l^{2} + h ^{2} } }}} \right)$$

**Fig. 6:**
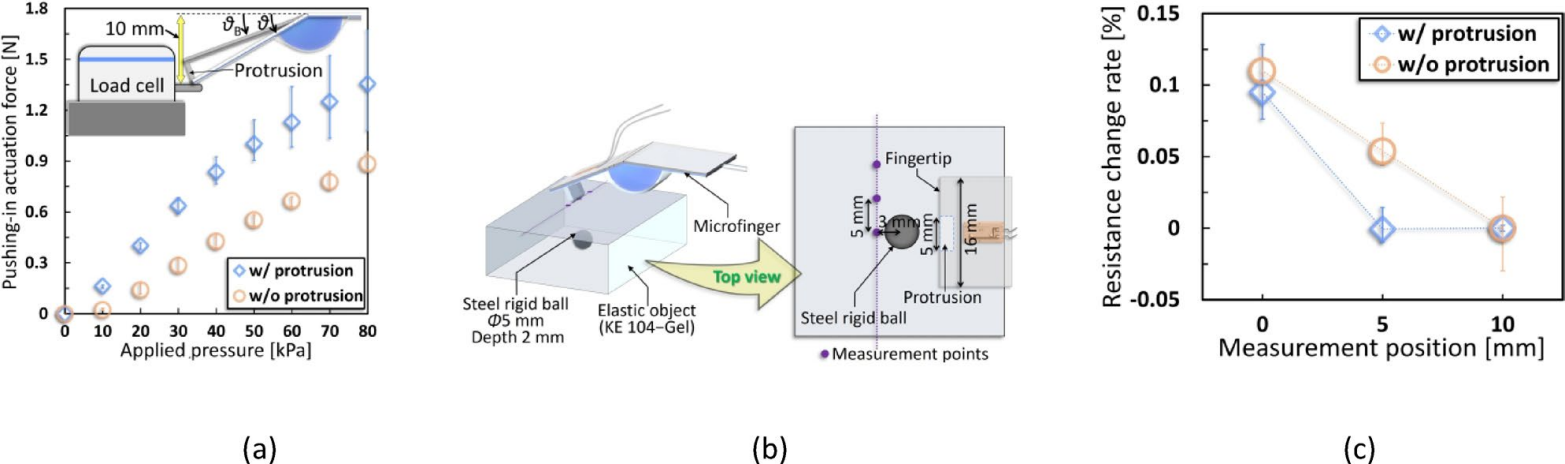
Performance improvement of the microfinger by protrusion integration (**a**) Our previous work reported the characteristics of the actuator used in the microfinger, and the generated force increased as the bending angle $\it {\uptheta }$ was smaller^21^. The force applied to the object was thought to be increased because the bending angle at the time of pushing-in was decreased by integrating the protrusion. (**b**) Resolution-evaluations were measured 3 mm in front of the rigid ball. Measurements were done in front of the rigid ball and at 3 points moved 5 mm intervals up to 10 mm horizontally. (**c**) Without protrusion, the rigid ball and microfinger did not overlap in the 10 mm position and partially overlapped in the 5 mm position. On the other hand, in the case of integrated protrusion, the protrusion and the rigid ball did not overlap at either the 5 and 10 mm positions, and the resistance change rate increased only at the 0 mm position.

Next, we evaluated the three-dimensional localizing algorithm using the protrusion-integrated microfinger based on the measurements illustrated in Fig. 3b. The measurement results corresponding to Fig. 3c, d are shown in Fig. 5d, e, respectively. As the integration of the protrusion structure increased the pushing-in actuation force (Fig. 6a) and improved the detection performance, the conditions (*D* = 12 and 15 mm) were evaluated in addition to *D* = 2, 5, 7, and 10 mm (Fig. 3c). In this evaluation, an increase in the elastic force was observed at the measurement points except for the ${\it \text{L}}_{{0}} {\text{ = 0 mm}}$ under each condition owing to the improved detection ability and enhanced directivity offered by the integration of the protrusion. As shown in Fig. 5d, the range of the resistance change rates differed for each depth condition, as the applied pressure differed for each condition (constant within the same trial) to avoid *T* > *D*; ${\it \text{L}}_{{0}} {\text{ = 0 mm}}$ was used as the standard. Therefore, our focus was not on comparing the different depth conditions but on the relative high/low relationship between the resistance change rates under the same conditions. As shown in Fig. 5d, the position where the elastic force increased moved to the right as *D* increased. Moreover, Fig. 5e shows that the theoretical and measured values were almost equivalent. Theoretically, the theoretical green plot was expected to coincide with the red plot representing the positions of the maximum force or toward the orange plot, which indicated the second-highest force peak. However, as shown in Fig. 5e, there were cases where the theoretical plot was positioned closer to the orange plot, which represent the second largest position (*D* = 12 and 15 mm), respectively, or where the arrangements of the plots were reversed, such as in the *D* = 2 and 15 mm cases. Based on this information, we assumed that there were errors in the depth-estimation (${\it\text{D}}_{{{\text{error}}}}$) obtained using this measurement. The causes of these errors included misalignment or deviations in the measured angles due to structural deformation, the detection accuracy of the microfinger, and measurement intervals, among other factors. However, based on the consideration of these factors, the accuracy of further depth-estimations was expected to improve.

### Improvement of the pushing-in actuation force by the protrusion structure

Here, we described the improvement of the palpation performance of the protrusion-integrated microfinger. The integrated protrusion is expected to improve the pushing-in actuation force, as stated in its design development. We have already reported the properties of the actuator used to design the microfinger^21^. Herein, we reported that the generated force decreased as $\it {\uptheta }$ increased. Thus, the pushing-in actuation force was assumed to increase with smaller $\it {\uptheta }$.

Figure 6a shows a comparison of the pushing-in actuation force with and without the protrusion. As observed, the distance between a load cell and the microfinger was fixed at 10 mm, and the pushing-in actuation force was measured. The measurement results (Fig. 6a) indicated that the protrusion-integrated microfinger increased the pushing-in actuation force. The error bar range of the with-protrusion case increased as the applied pressure increased. This might be caused by a large deformation of the microfinger structure as the pushing-in actuation force increased, causing losses in force conversion and inducing large individual differences. Additionally, the height of the protrusion was set at 5 mm in this case, although we considered the case when it was even higher. It can be assumed that the pushing-in actuation force increased as the protrusion became higher (maximum height ≤ $\it {\text{u}}$) at a constant $\it {\text{u}}$. However, $\it {\uptheta }$ also decreased concurrently, making the direction of the pushing-in force more vertical, thus probably decreasing in directivity. Therefore, the protrusion height must be designed considering the trade-off between pushing-in actuation force and directivity.

### Resolution improvement by the integrated protrusion structure

The relationship between protrusion contact width and resolution is described. The microfinger detects increased elastic force due to a singularity along the pushing-in direction; however, detection requires the contact area to overlap the singularity. The two-dimensional localization algorithm (Fig. 1b) involves measurements at regular intervals along axes defined relative to the singularity. The minimum interval required for detection depends on the singularity size^18^, and localization accuracy improves with decreasing interval width. However, using a microfinger with a wide contact width at small intervals may cause the singularity to be detected at multiple nearby points, reducing the accuracy of position estimation. This highlights the need to enhance the detection resolution of the microfinger.

Figure 6b illustrates the resolution evaluation method using an integrated protrusion with a narrow contact width. Measurements were taken at three points spaced 5 mm apart along the singularity, following the same approach as the two-dimensional localization algorithm. The depth of the rigid ball (*D*) was set to 2 mm, and the measurement axis was placed 3 mm in front of the point directly over the singularity, based on the results in Figs. 3c and 5d. The contact width with the protrusion was 5 mm (Fig. 4b), while it was 16 mm without the protrusion.

Figure 6c presents the evaluation results. The measurement point in front of the rigid ball was defined as 0 mm, and measurements were taken at horizontal intervals of 5 mm up to 10 mm. The vertical and horizontal axes represent resistance change rate and measurement position, respectively, with the resistance change rate normalized to the value at the 10 mm position. At 5 mm, the microfinger with the 5 mm-wide protrusion did not detect the rigid ball due to lack of overlap, whereas the 16 mm-wide version without the protrusion partially overlapped the ball and detected it. These results indicate that narrowing the contact width improves resolution and enables more accurate localization at smaller intervals. However, a narrower contact width reduces scanning efficiency, as it covers a smaller area per scan. Therefore, the protrusion width should be designed by balancing accuracy and efficiency according to application requirements.

### Depth-estimation accuracy

**Fig. 7:**
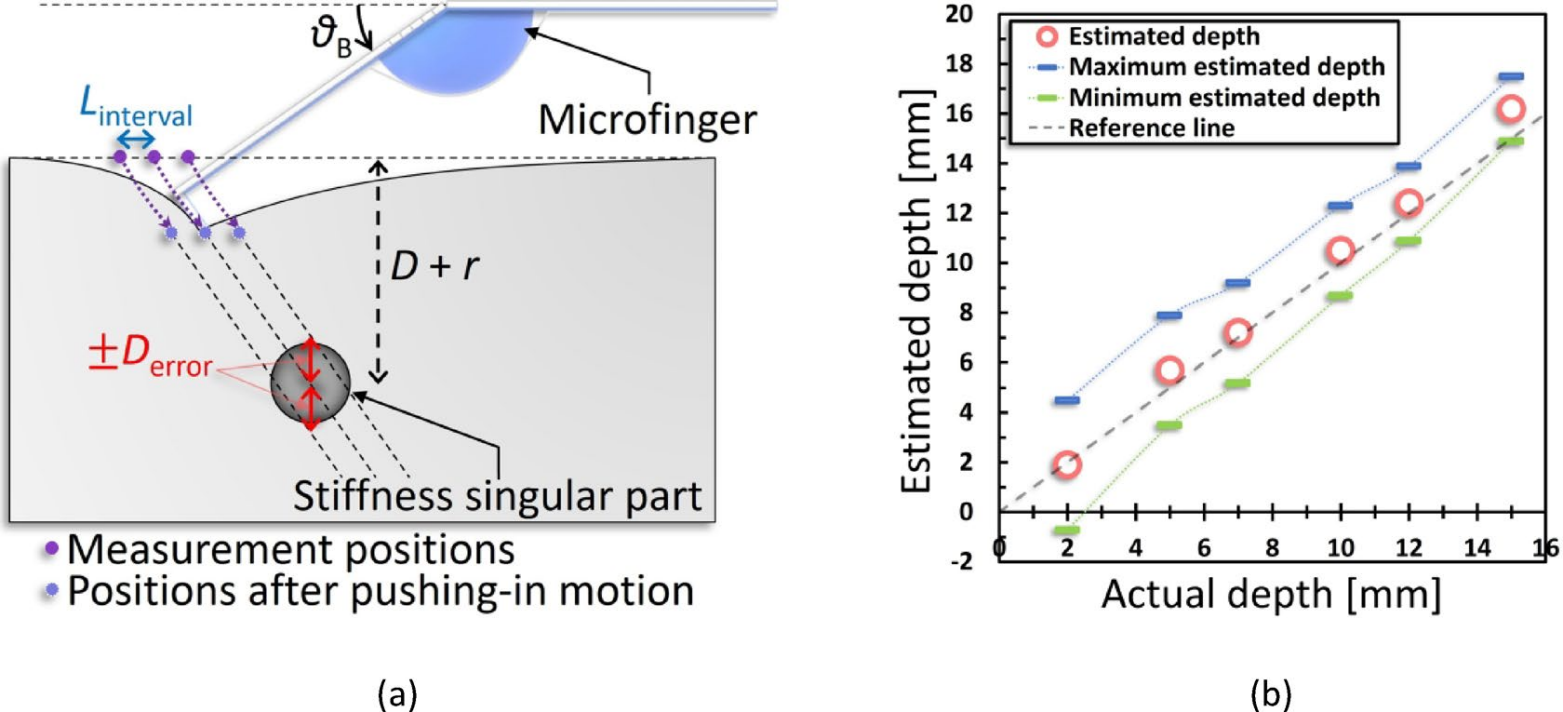
Measuring Interval and depth-estimation error (**a**) The error in the depth-estimation accuracy of the singularity improves with decreasing measurement intervals. As shown in Fig. 5a, the singularity was considered to exist within the three highest points of the measured values. Therefore, it is thought that the error in the estimated depth decreases by narrowing the measurement interval. (**b**) Estimated versus actual depths and corresponding errors for each condition, based on Fig. 5e.

The depth-estimation accuracy based on the three-dimensional localizing algorithm (Fig. 5e) may have been affected by errors due to misalignment, structural deformation, microfinger-detection precision, and measurement intervals. In particular, the estimation error is expected to increase further due to surface irregularities and internal structural variations when palpating actual organs. To address this, a method that determines depth comprehensively based on the results from multiple measurement points would be necessary. Thus, we evaluated the estimation accuracy of depth with respect to measurement intervals. Figure 7a shows the relationship between the measurement interval and ${\it \text{D}}_{{{\text{error}}}}$. The singularity may have probably existed inside the three extensions of the pushing-in direction at the three measurement points with the highest elastic-force increase (Fig. 5e). Here, ${\it\text{D}}_{{{\text{error}}}}$ can be calculated using the measurement interval ${\it \text{L}}_{{{\text{interval}}}}$, as follows:

14 $$\mathit{D}_{\mathrm{error}} = \frac{\mathit{L}_{\mathrm{interval}}}{\tan \mathit{\theta}_{\mathrm{B}}}$$

Equation 14 reveals that ${\it\text{D}}_{{{\text{error}}}}$ decreased as ${\it\text{L}}_{{{\text{interval}}}}$ decreased. Moreover, ${\it\text{D}}_{{{\text{error}}}}$ in Fig. 5e was derived using the equation. The estimated depths from the results of Fig. 5e and the errors derived from Eq. 14 are depicted in Fig. 7b. In this evaluation, measurements were conducted at 1 mm intervals, and the theoretical value plots were within the range of the first to third-largest plots in Fig. 5e. The value of ${{{\it \uptheta }}}_{{\text{B}}}$ used in each trial was the one measured during the experiment. As shown in Fig. 7b, the estimated depths fell within a few millimeters of error, and the error decreased for deeper targets. Although shallow targets resulted in larger errors, we believe this can be addressed by adjusting the conditions of ${\it\text{L}}_{{{\text{interval}}}}$ and ${{{\it\uptheta }}}_{{\text{B}}}$. In the case of *D* = 15 mm, the estimated depth began to deviate from the actual depth, suggesting that measurement errors due to alignment and other factors become more significant for deeper targets. The maximum detectable depth is considered to strongly depend on factors such as the elasticity of the target, sensor sensitivity, pushing-in force, and the design of the protrusion, and future evaluations using biological tissue are necessary.

## Materials and methods

### Fabrication of the microfinger

The microfinger (16 mm × 40 mm × 965 µm) was fabricated by combining an inflatable bending microactuator^21,22^ (comprising PBA as an artificial muscle and a conversion film) with a piezoresistive strain sensor comprising a polyimide base film (4.0 mm × 7.7 mm; KSPB-2-1 K-E4, Kyowa Electronic Instruments Co., Ltd.). The diameter of the balloon in PBA was 13 mm, and it was fabricated by bonding a thin silicone-rubber film (KE-1606, Shin-Etsu Chemical Co., Ltd.)^21,22^. The surface of the balloon was coated with parylene (thickness: 0.1 µm; Parylene C, Specialty Coating Systems Inc.) to seal and reduce the friction coefficient with the conversion film. The balloon patterns were transferred by spin-coating KE-1606 onto an SU-8 mold formed on Si wafers. The base structure (Fig. 3a) was made of amorphous polyethylene terephthalate (A-PET, thickness: 500 μm). The PET film (Lumirror T60, Toray Industries, Inc.) and KE-1606 were used as the conversion films. A silane coupling agent (3-mercaptopropyltrimethoxysilane) was used for the PET film, which was bonded to the KE-1606 thin film (KE-1606 was spin-coated at 2000 rpm on the Si wafer and thermally cured for 10 min at 85 °C). Thereafter, KE-1606 bonded to the PET film surface was bonded to PBA via surface activation (vacuum ultraviolet light). Further, a double-sided tape to adhere the silicone rubber (Nitto Denko Corporation.) was used to adhere the PBA and base structure.

As shown in Fig. 4b, the protrusion was made by covering a silicone rubber cap (thickness: 0.5 mm; KE-1300 series, Shin-Etsu Chemical Co., Ltd.) to a polycarbonate (4 mm × 4.5 mm × 1 mm) as the core.

### Preparation for the elastic objects encapsulating the rigid ball

The evaluation samples were prepared using a rigid steel ball (diameter: 5 mm) and silicone gel (KE-104 Gel, Shin-Etsu Chemical Co., Ltd.) as the singularity and elastic material, respectively. The mixing ratio of the prepolymer to the KE-104 Gel curing agent was 10:1, and Young’s modulus, which was measured by a commercialized elasticity measurement instrument (YAWASA, YWS-5N-1) was approximately 5 kPa. Furthermore, KE-104 Gel was cured (150 °C, 1 h) in a petri dish (stainless steel petri dish (SUS304), SUNDIA Corporation; Φ120 mm × 25 mm) to encapsulate the rigid ball. A thin PDMS coat (SILPOT®184 W/C, Dow Corning Toray Co., Ltd.) was subsequently applied to the surface to improve the strength and evenness of the KE-104 Gel surface as well as suppress adhesion; this was followed by curing (80 °C, 20 min).

### Experimental setup

To evaluate the three-dimensional localizing algorithm and resolution, the microfinger was fixed to the jig and positioned by the Z-stage. Thereafter, the measurement sample was fixed on the XY-stage for positioning. Pressure was applied to the microfinger using a pressure-control system. The resistance values of the strain sensor were measured using a digital multimeter (DMM6500, Keithley Instruments). Moreover, the bending angle of the microfinger was measured using a digital angle meter (Bevel Box BB-01B, AS ONE Corporation.).

For the force measurement, a load cell (LVS-500GA; Kyowa Electronic Instruments Co., Ltd.) and a strain amplifier (DPM-911B; Kyowa Electronic Instruments Co., Ltd.) were used.

## Conclusions

We report depth-estimation of stiffness singularities within elastic objects based on directional touch sensing using a strain-sensor-integrated microfinger with directivity. Evaluation results confirmed that the proposed technology estimated the depth. By integrating a protrusion on the microfinger’s contact part, we estimated deeper singularities. We also confirmed that the protrusion improves pushing-in actuation force and resolution. Depth-estimation accuracy can be further improved by reducing measurement intervals. Overall, we developed a protrusion-integrated microfinger for evaluating the stiffness of soft objects. We also applied this microfinger to the detection of stiffness singularities within elastic objects, thus demonstrating its application potential in searching for tumors, which exist as singularities in elastic organs. Our technology can advance palpation-based diagnostic techniques in scope-assisted surgery.

## Data Availability

All data generated or analyzed during this study are included in this published article. The raw datasets are available from the corresponding authors, Y.H. and S.K., upon reasonable request.
